# Supplementary figures and images for: Proximity labeling identifies LOTUS domain proteins that promote the formation of perinuclear germ granules in C. elegans
Source: eLife. 2021 Nov 3;10:e72276. doi: 10.7554/eLife.72276 (PMC8616582; doi:10.7554/eLife.72276)

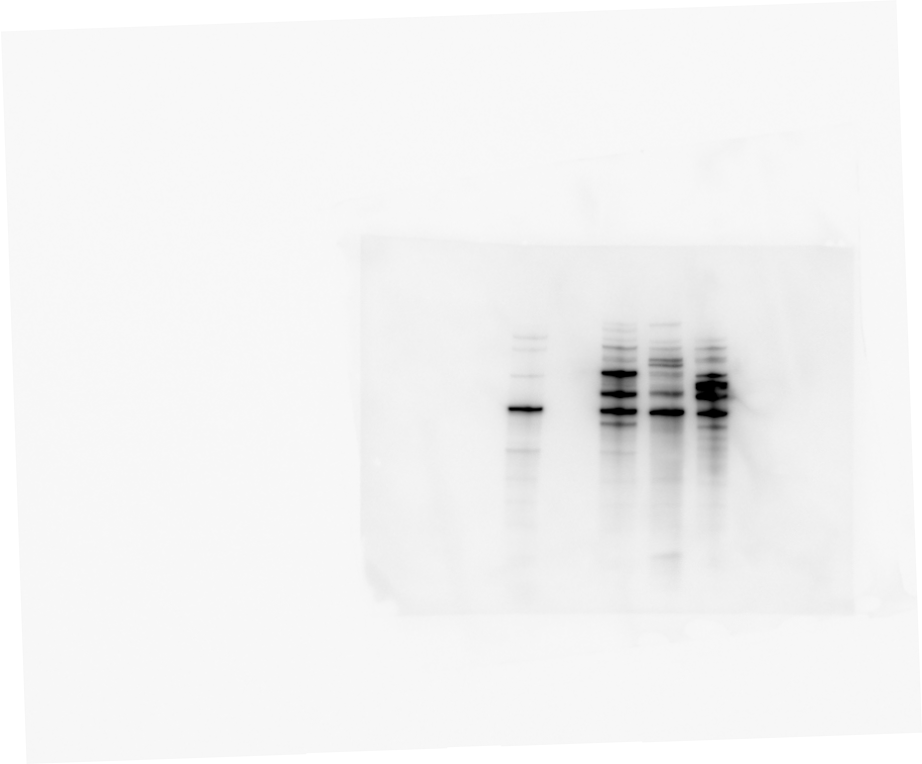

Supplement: Figure 1—source data 2. [file elife-72276-fig1-data2.zip › Figure 1-Source Data 2-uncropped_blot_images/Figure 1-Source Data 2- streptavidin HRP blot of whole-animal lysates.tif]

Figure 1 source data

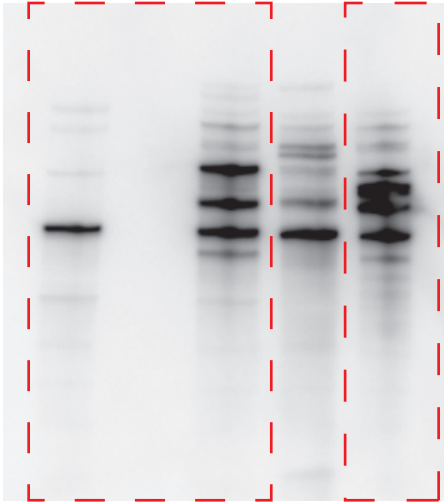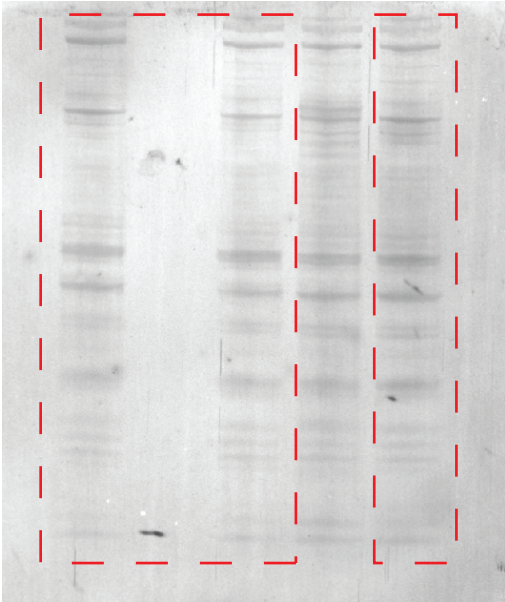

Supplement: Figure 1—source data 2. [file elife-72276-fig1-data2.zip › Figure 1-Source Data 2-uncropped_blot_images/Figure 1_labeled.pdf]

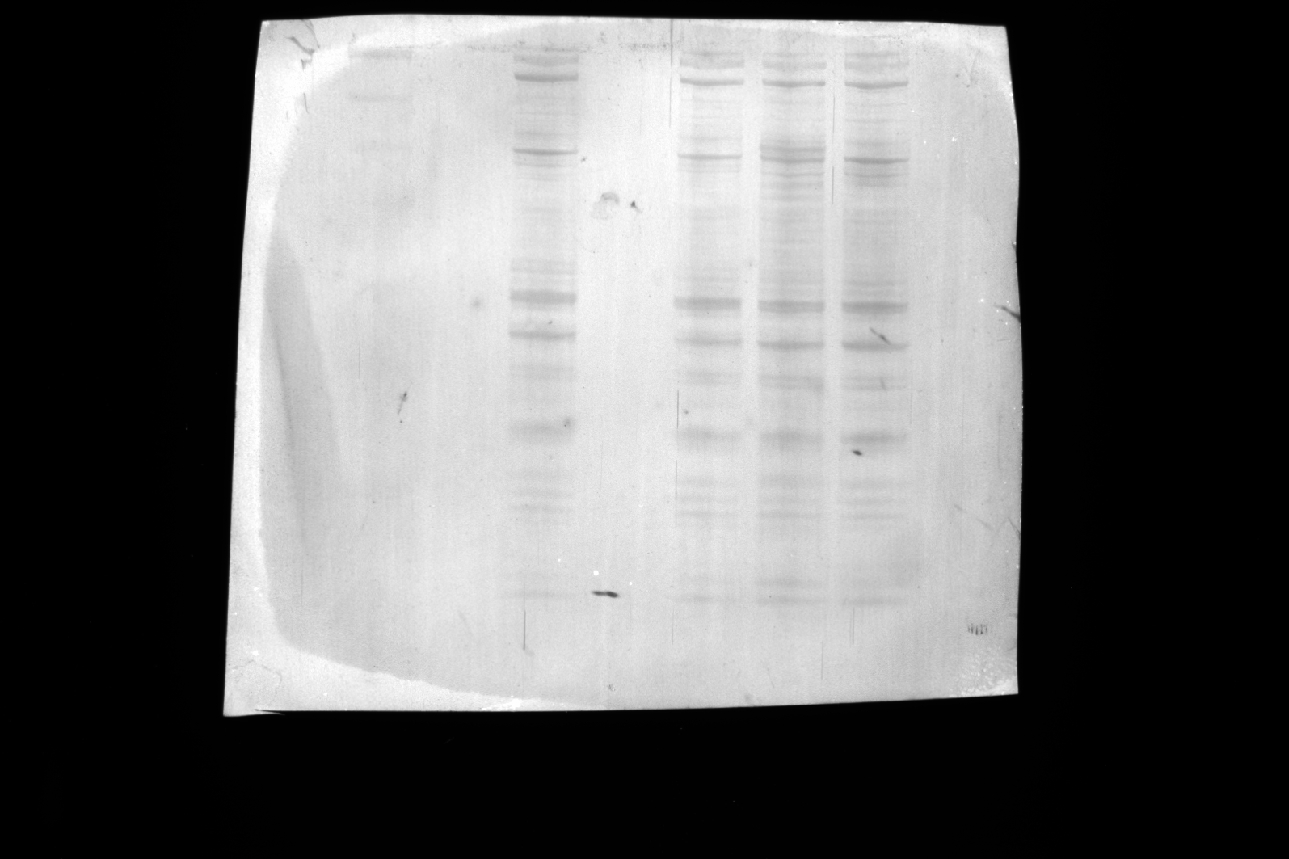

Supplement: Figure 1—source data 2. [file elife-72276-fig1-data2.zip › Figure 1-Source Data 2-uncropped_blot_images/Figure 1-Source Data 2- coomassie blue staining of streptavidin blot membrane.jpg]

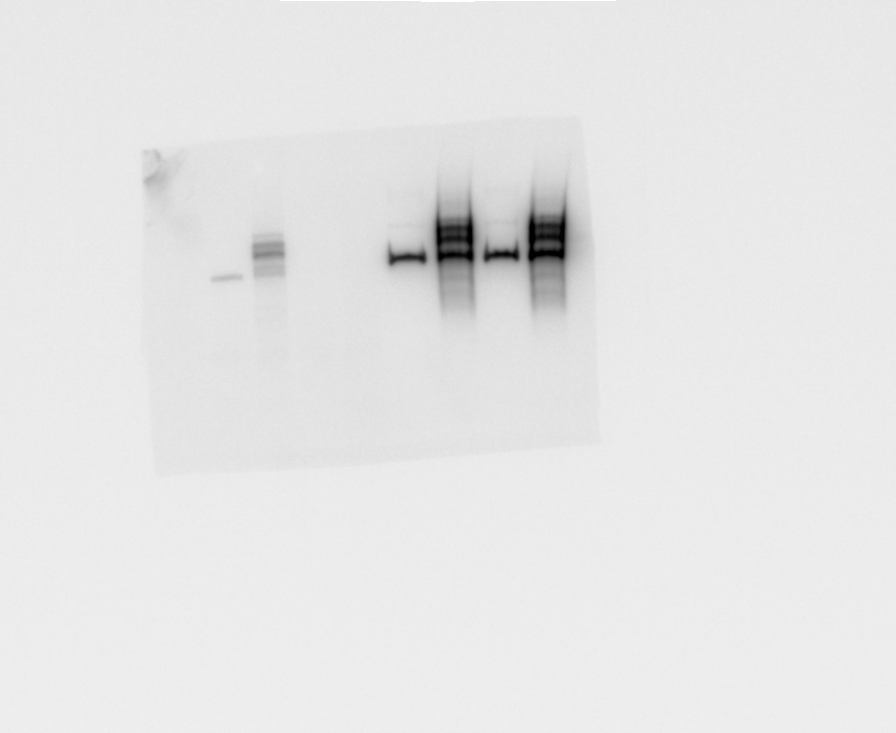

Supplement: Figure 1—figure supplement 1—source data 1. [file elife-72276-fig1-figsupp1-data1.zip › Figure 1ΓÇöfigure supplement 1-Source data 1/Figure 1ΓÇöfigure supplement 1-streptavidin HRP blot of biotynlated input, IP, and flow-through.tif]

Figure 1—figure supplement 1 Source data

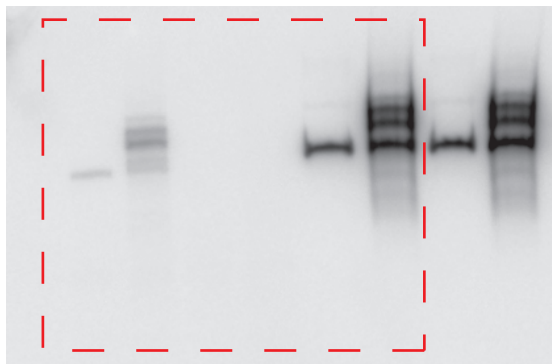

Supplement: Figure 1—figure supplement 1—source data 1. [file elife-72276-fig1-figsupp1-data1.zip › Figure 1ΓÇöfigure supplement 1-Source data 1/Figure 1ΓÇöfigure supplement 1-Source data 1_labled.pdf]
