# Supplementary material for: Proximity labeling identifies LOTUS domain proteins that promote the formation of perinuclear germ granules in C. elegans
Source: eLife. 2021 Nov 3;10:e72276. doi: 10.7554/eLife.72276 (PMC8616582; doi:10.7554/eLife.72276)
Supplement: Transparent reporting form [file elife-72276-transrepform1.docx]

***eLife’s* transparent reporting form**

We encourage authors to provide detailed information *within their submission* to facilitate the interpretation and replication of experiments. Authors can upload supporting documentation to indicate the use of appropriate reporting guidelines for health-related research (see [EQUATOR Network](http://www.equator-network.org/%20)), life science research (see the [BioSharing Information Resource](https://biosharing.org/)), or the [ARRIVE guidelines](http://www.plosbiology.org/article/info:doi/10.1371/journal.pbio.1000412) for reporting work involving animal research. Where applicable, authors should refer to any relevant reporting standards documents in this form.

If you have any questions, please consult our Journal Policies and/or contact us: [editorial@elifesciences.org](mailto:editorial@elifesciences.org).

**Sample-size estimation**

- You should state whether an appropriate sample size was computed when the study was being designed
- You should state the statistical method of sample size computation and any required assumptions
- If no explicit power analysis was used, you should describe how you decided what sample (replicate) size (number) to use

Please outline where this information can be found within the submission (e.g., sections or figure legends), or explain why this information doesn’t apply to your submission:

Sample sizes are indicated in figure legends or methods. No explicit power analysis was used. Sample sizes were determined based on qualitative variability of data and practical constraints based on techniques used.

**Replicates**

- You should report how often each experiment was performed
- You should include a definition of biological versus technical replication
- The data obtained should be provided and sufficient information should be provided to indicate the number of independent biological and/or technical replicates
- If you encountered any outliers, you should describe how these were handled
- Criteria for exclusion/inclusion of data should be clearly stated
- High-throughput sequence data should be uploaded before submission, with a private link for reviewers provided (these are available from both GEO and ArrayExpress)

Please outline where this information can be found within the submission (e.g., sections or figure legends), or explain why this information doesn’t apply to your submission:

Streptavidin staining images are representative of experiments carried out at least 3 times, with at least 3 biological replicates each. RNAi phenotypes for RNAi screen are summaries of at least 4 separate animals from the same RNAi condition. For all other experiments, number of replicates are indicated in the figure legends.

**Statistical reporting**

- Statistical analysis methods should be described and justified
- Raw data should be presented in figures whenever informative to do so (typically when N per group is less than 10)
- For each experiment, you should identify the statistical tests used, exact values of N, definitions of center, methods of multiple test correction, and dispersion and precision measures (e.g., mean, median, SD, SEM, confidence intervals; and, for the major substantive results, a measure of effect size (e.g., Pearson's r, Cohen's d)
- Report exact p-values wherever possible alongside the summary statistics and 95% confidence intervals. These should be reported for all key questions and not only when the p-value is less than 0.05.

Please outline where this information can be found within the submission (e.g., sections or figure legends), or explain why this information doesn’t apply to your submission:

Mean values for IP enrichment are shown in Figure 2A. All other figures show raw data values. Precise numbers of N are indicated in the figure or figure legend for all graphs. Statistical tests used are described either in the Methods or figure legends.

(For large datasets, or papers with a very large number of statistical tests, you may upload a single table file with tests, Ns, etc., with reference to sections in the manuscript.)

**Group allocation**

- Indicate how samples were allocated into experimental groups (in the case of clinical studies, please specify allocation to treatment method); if randomization was used, please also state if restricted randomization was applied
- Indicate if masking was used during group allocation, data collection and/or data analysis

Please outline where this information can be found within the submission (e.g., sections or figure legends), or explain why this information doesn’t apply to your submission:

No group allocation was performed.

**Additional data files (“source data”)**

- We encourage you to upload relevant additional data files, such as numerical data that are represented as a graph in a figure, or as a summary table
- Where provided, these should be in the most useful format, and they can be uploaded as “Source data” files linked to a main figure or table
- Include model definition files including the full list of parameters used
- Include code used for data analysis (e.g., R, MatLab)
- Avoid stating that data files are “available upon request”

Please indicate the figures or tables for which source data files have been provided:

Source data for Figure 1B are present in Figure 1-Source Data 1. Source data for Figure 2A, are provided in Figure 2-Source Data 1. Uncropped blots of Figure 1C are included in Figure 1-Source Data 2. Uncropped blots of Figure 1—figure supplement 1B are included in Figure 1-figure supplement 1-Source data 1. Source data for Figure 2D, 2E, and 2F are present in supplemental tables S1 and S2. Random control groups in Figure 2E and 2F are sampled from Figure 2- Source Data 2. Source data for Figure 3A, 3E, and 3F are provided in Figure 3- Source Data 1, Figure 3- Source Data 2, and Figure 3-Source Data 3, respectively. Source Data for Figure 3D are provided in Supplementary File 4. Source Data for Figure 4A+4B and 4E are provided in Figure 4-Source Data 1, Figure 4-Source Data 2, respectively. Source data for Figure 3-Supplement 1 are provided in Figure 3-Supplement 1-Source Data 1. Code used for analyses are provided through the github link in the Methods section (https://github.com/benpastore/TurboID).
